# {2,2-Bis[(4*S*)-4-*tert*-butyl-4,5-dihydro-1,3-oxazol-2-yl]propane}bis­(*N*,*N*-di­methyl­formamide)copper(II) bis­(hexa­fluoridoantimonate)

**DOI:** 10.1107/S1600536809020364

**Published:** 2009-06-06

**Authors:** Julia Rehbein, Markus Schürmann, Hans Preut, Martin Hiersemann

**Affiliations:** a Fakultät Chemie, Technische Universität Dortmund, Otto-Hahn-Strasse 6, 44221 Dortmund, Germany

## Abstract

In the title compound, [Cu(C_17_H_30_N_2_O_2_)(C_3_H_7_NO)_2_][SbF_6_]_2_, which is a potential catalyst in the asymmetric Gosteli–Claisen rearrangement, the Cu atom adopts a distorted *cis*-CuN_2_O_2_ square-planar geometry arising from *N*,*N*′-bidentate coordin­ation by the chiral ligand and two O-bonded dimethyl­formamide mol­ecules. Two short C—H⋯O contacts occur within the ligand and two weak inter­molecular C—H⋯F bonds may help to establish the packing.

## Related literature

For further synthetic details, see: Evans *et al.* (1981 ▶, 1999 ▶); Meyers & McKennon (1993 ▶). For information on the catalytic properties of the title compound, see: Abraham *et al.* (2001 ▶, 2004 ▶); Abraham & Hiersemann (2001 ▶); Hiersemann & Abraham (2002 ▶).
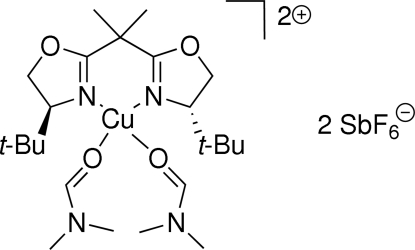


## Experimental

### 

#### Crystal data

[Cu(C_17_H_30_N_2_O_2_)(C_3_H_7_NO)_2_][SbF_6_]_2_
*M* *_r_* = 975.66
Monoclinic, 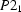

*a* = 9.1550 (5) Å
*b* = 13.6852 (8) Å
*c* = 14.5359 (8) Å
β = 92.570 (5)°
*V* = 1819.34 (18) Å^3^
*Z* = 2
Mo *K*α radiation
μ = 2.15 mm^−1^
*T* = 173 K
0.18 × 0.12 × 0.04 mm

#### Data collection

Oxford Diffraction Xcalibur-S CCD diffractometer
Absorption correction: multi-scan (*CrysAlis RED*; Oxford Diffraction, 2008 ▶) *T*_min_ = 0.808, *T*_max_ = 1.000 (expected range = 0.741–0.918)
10322 measured reflections
6226 independent reflections
4042 reflections with *I* > 2σ(*I*)
*R*_int_ = 0.037

#### Refinement

*R*[*F* ^2^ > 2σ(*F* ^2^)] = 0.038
*wR*(*F* ^2^) = 0.048
*S* = 0.85
6226 reflections
407 parameters
1 restraint
H-atom parameters constrained
Δρ_max_ = 0.96 e Å^−3^
Δρ_min_ = −0.67 e Å^−3^
Absolute structure: Flack (1983 ▶), 2689 Friedel pairs
Flack parameter: 0.015 (15)

### 

Data collection: *CrysAlis CCD* (Oxford Diffraction, 2008 ▶); cell refinement: *CrysAlis CCD*; data reduction: *CrysAlis RED* (Oxford Diffraction, 2008 ▶); program(s) used to solve structure: *SHELXS97* (Sheldrick, 2008 ▶); program(s) used to refine structure: *SHELXL97* (Sheldrick, 2008 ▶); molecular graphics: *SHELXTL-Plus* (Sheldrick, 2008 ▶); software used to prepare material for publication: *SHELXL97* and *PLATON* (Spek, 2009 ▶).

## Supplementary Material

Crystal structure: contains datablocks I, global. DOI: 10.1107/S1600536809020364/hb2975sup1.cif

Structure factors: contains datablocks I. DOI: 10.1107/S1600536809020364/hb2975Isup2.hkl

Additional supplementary materials:  crystallographic information; 3D view; checkCIF report

## Figures and Tables

**Table:** 

Cu—N1	1.929 (5)
Cu—N2	1.919 (5)
Cu—O11	1.926 (4)
Cu—O12	1.951 (4)

**Table:** 

N2—Cu—O11	153.82 (19)
N2—Cu—N1	92.92 (17)
O11—Cu—N1	91.37 (19)
N2—Cu—O12	95.00 (19)
O11—Cu—O12	91.51 (17)
N1—Cu—O12	155.84 (18)

**Table 2:** Hydrogen-bond geometry (Å, °)

*D*—H⋯*A*	*D*—H	H⋯*A*	*D*⋯*A*	*D*—H⋯*A*
C12—H12*A*⋯O11	0.98	2.58	3.204 (8)	122
C17—H17*C*⋯O12	0.98	2.58	3.189 (7)	120
C25—H25*C*⋯F13^i^	0.98	2.55	3.418 (8)	148
C26—H26*B*⋯F11^i^	0.98	2.53	3.427 (8)	152

Symmetry code: (i) 
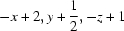
.

**Fig. 1.**
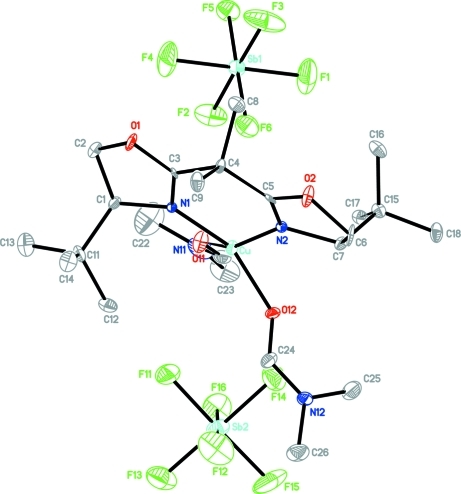
The molecular structure of (I) with displacement ellipsoids shown at the 30% probability level.

## supplementary crystallographic information

### Comment

The title compound, (I), was tested as a catalyst in the catalytic asymmetric Gosteli-Claisen rearrangement (Abraham *et al.*, 2001; Abraham & Hiersemann, 2001; Hiersemann & Abraham, 2002; Abraham *et al.*, 2004). The synthesis of the title compound, (I), was accomplished according to a modified procedure of Evans *et al.* (1999). A sequence of Meyer's amino acid reduction of (*S*)-*tert*-Leucine (Meyers & McKennon, 1993), subsequent condensation with dimethyl malonic acid dichloride and *p*-TsCl catalyzed cyclization provided the (*S,S*)-*t*-Bu-box ligand. Treatment of the box ligand with CuCl_2_ (Evans *et al.*, 1981) and subsequent anion metathesis with AgSbF_6_ provided [Cu(*S,S*)-*t*-Bu-box](SbF_6_)_2_. Addition of 2 eq of DMF to a solution of [Cu(*S,S*)-*t*-Bu-box](SbF_6_)_2_ in 1,2-dichloroethane afforded the bis(*N*,*N*-dimethylformamide) complex. Crystallization was achieved by vapor diffusion recrystalization at 243 K.

### Experimental

To a solution of [Cu(*S,S*)-*t*-Bu-box](SbF_6_)_2 _(15.4 mg, 17.3 mmol, 1 eq) in dry 1,2-dichloroethane (1 ml) under argon atmosphere, DMF (27 µ*L*, 34.6 mmol, 2eq) was added by a microliter syringe and the resulting deep blue solution was stirred for 30 min at room temperature. Subsequent cooling to 243 K for 24 h provided (I) as deep blue crystals. IR (from a 0.05 *M* 1,2-dichloroethane solution, cm^-1^): 1750 (w), 1680 (*s*); UV/vis (from a 0.05 *M* in 1,2-dichloroethane solution, nm): 255, 733.

### Crystal data

**Table:**

[Cu(C_17_H_30_N_2_O_2_)(C_3_H_7_NO)_2_][SbF_6_]_2_	*F*(000) = 962
*M**_r_* = 975.66	*D*_x_ = 1.781 Mg m^−^^3^
Monoclinic, *P*2_1_	Mo *K*α radiation, λ = 0.71073 Å
Hall symbol: P 2yb	Cell parameters from 3964 reflections
*a* = 9.1550 (5) Å	θ = 2.1–29.0°
*b* = 13.6852 (8) Å	µ = 2.15 mm^−^^1^
*c* = 14.5359 (8) Å	*T* = 173 K
β = 92.570 (5)°	Plate, blue
*V* = 1819.34 (18) Å^3^	0.18 × 0.12 × 0.04 mm
*Z* = 2	

### Data collection

**Table:**

Oxford Diffraction Xcalibur-S CCD diffractometer	6226 independent reflections
Radiation source: fine-focus sealed tube	4042 reflections with *I* > 2σ(*I*)
graphite	*R*_int_ = 0.037
Detector resolution: 16.0560 pixels mm^-1^	θ_max_ = 25.5°, θ_min_ = 2.0°
ω scans	*h* = −11→10
Absorption correction: multi-scan (*CrysAlis RED*; Oxford Diffraction, 2008)	*k* = −16→16
*T*_min_ = 0.808, *T*_max_ = 1.000	*l* = −17→17
10322 measured reflections	

### Refinement

**Table:**

Refinement on *F*^2^	Secondary atom site location: difference Fourier map
Least-squares matrix: full	Hydrogen site location: inferred from neighbouring sites
*R*[*F*^2^ > 2σ(*F*^2^)] = 0.038	H-atom parameters constrained
*wR*(*F*^2^) = 0.048	*w* = 1/[σ^2^(*F*_o_^2^) + (0.0061*P*)^2^] where *P* = (*F*_o_^2^ + 2*F*_c_^2^)/3
*S* = 0.85	(Δ/σ)_max_ = 0.001
6226 reflections	Δρ_max_ = 0.96 e Å^−^^3^
407 parameters	Δρ_min_ = −0.67 e Å^−^^3^
1 restraint	Absolute structure: Flack (1983), 2689 Friedel pairs
Primary atom site location: structure-invariant direct methods	Flack parameter: 0.015 (15)

### Special details

**Table:**

Experimental. Absorption correction: CrysAlis RED (Oxford Diffraction 2008) Empirical absorption correction using spherical harmonics, implemented in SCALE3 ABSPACK scaling algorithm.
Geometry. All e.s.d.'s (except the e.s.d. in the dihedral angle between two l.s. planes) are estimated using the full covariance matrix. The cell e.s.d.'s are taken into account individually in the estimation of e.s.d.'s in distances, angles and torsion angles; correlations between e.s.d.'s in cell parameters are only used when they are defined by crystal symmetry. An approximate (isotropic) treatment of cell e.s.d.'s is used for estimating e.s.d.'s involving l.s. planes.
Refinement. Refinement of *F*^2^ against ALL reflections. The weighted *R*-factor *wR* and goodness of fit *S* are based on *F*^2^, conventional *R*-factors *R* are based on *F*, with *F* set to zero for negative *F*^2^. The threshold expression of *F*^2^ > σ(*F*^2^) is used only for calculating *R*-factors(gt) *etc*. and is not relevant to the choice of reflections for refinement. *R*-factors based on *F*^2^ are statistically about twice as large as those based on *F*, and *R*- factors based on ALL data will be even larger.

### Fractional atomic coordinates and isotropic or equivalent isotropic displacement parameters (Å^2^)

**Table:**

	*x*	*y*	*z*	*U*_iso_*/*U*_eq_	
Sb1	0.24602 (5)	−0.00096 (4)	0.84532 (3)	0.02849 (13)	
Sb2	0.82483 (6)	−0.04083 (3)	0.35372 (3)	0.04390 (17)	
Cu	0.73163 (7)	0.00310 (7)	0.75133 (4)	0.01763 (19)	
F1	0.2031 (5)	0.1198 (3)	0.7920 (4)	0.0916 (18)	
F2	0.2546 (4)	−0.0541 (4)	0.7284 (3)	0.0698 (15)	
F3	0.2369 (5)	0.0546 (4)	0.9607 (3)	0.101 (2)	
F4	0.2858 (6)	−0.1211 (3)	0.8965 (3)	0.0863 (18)	
F5	0.0475 (3)	−0.0245 (3)	0.8405 (2)	0.0477 (12)	
F6	0.4443 (4)	0.0246 (3)	0.8467 (2)	0.0509 (14)	
F11	0.7475 (5)	−0.1293 (3)	0.4335 (3)	0.0786 (18)	
F12	0.9927 (4)	−0.0336 (4)	0.4310 (3)	0.0952 (17)	
F13	0.9126 (5)	−0.1425 (4)	0.2906 (3)	0.0789 (17)	
F14	0.7367 (5)	0.0599 (3)	0.4194 (3)	0.0686 (16)	
F15	0.9021 (6)	0.0505 (4)	0.2763 (3)	0.099 (2)	
F16	0.6569 (4)	−0.0534 (4)	0.2775 (3)	0.0743 (14)	
O1	0.6721 (5)	−0.1668 (3)	0.9773 (3)	0.0260 (12)	
O2	0.8559 (4)	0.1567 (3)	0.9822 (3)	0.0268 (12)	
O11	0.6003 (4)	−0.0630 (3)	0.6648 (3)	0.0260 (12)	
O12	0.8222 (5)	0.0741 (3)	0.6524 (3)	0.0201 (11)	
N1	0.7133 (5)	−0.1001 (3)	0.8405 (3)	0.0103 (13)*	
N2	0.7798 (5)	0.0988 (3)	0.8442 (3)	0.0119 (13)*	
N11	0.4549 (6)	−0.0757 (5)	0.5361 (4)	0.0431 (19)	
N12	1.0155 (6)	0.1349 (4)	0.5795 (4)	0.0252 (14)	
C1	0.6719 (7)	−0.2052 (4)	0.8185 (4)	0.0171 (16)	
H1A	0.5896	−0.2058	0.7709	0.020*	
C2	0.6141 (7)	−0.2378 (5)	0.9112 (4)	0.0237 (19)	
H2A	0.5059	−0.2375	0.9091	0.028*	
H2B	0.6490	−0.3044	0.9274	0.028*	
C3	0.7127 (7)	−0.0906 (5)	0.9287 (4)	0.0148 (16)*	
C4	0.7635 (6)	−0.0028 (5)	0.9871 (3)	0.0161 (13)	
C5	0.7986 (7)	0.0843 (5)	0.9312 (4)	0.0178 (16)*	
C6	0.9016 (7)	0.2313 (4)	0.9176 (4)	0.0249 (18)	
H6A	1.0080	0.2276	0.9086	0.030*	
H6B	0.8772	0.2976	0.9394	0.030*	
C7	0.8128 (7)	0.2058 (4)	0.8278 (4)	0.0157 (16)	
H7A	0.8781	0.2113	0.7747	0.019*	
C8	0.6355 (7)	0.0246 (4)	1.0491 (4)	0.030 (2)	
H8A	0.5539	0.0509	1.0108	0.045*	
H8B	0.6689	0.0740	1.0942	0.045*	
H8C	0.6032	−0.0338	1.0815	0.045*	
C9	0.8971 (6)	−0.0356 (5)	1.0481 (3)	0.0277 (17)	
H9A	0.9769	−0.0540	1.0090	0.042*	
H9B	0.8700	−0.0918	1.0855	0.042*	
H9C	0.9289	0.0183	1.0886	0.042*	
C11	0.7994 (8)	−0.2652 (5)	0.7835 (4)	0.0230 (18)	
C12	0.8609 (7)	−0.2170 (5)	0.6982 (4)	0.0296 (19)	
H12A	0.7815	−0.2061	0.6519	0.044*	
H12B	0.9346	−0.2599	0.6726	0.044*	
H12C	0.9059	−0.1543	0.7156	0.044*	
C13	0.7361 (8)	−0.3652 (5)	0.7570 (5)	0.045 (2)	
H13A	0.6503	−0.3566	0.7151	0.067*	
H13B	0.7074	−0.3995	0.8126	0.067*	
H13C	0.8101	−0.4036	0.7264	0.067*	
C14	0.9207 (7)	−0.2766 (5)	0.8565 (4)	0.0298 (19)	
H14A	0.9925	−0.3239	0.8357	0.045*	
H14B	0.8798	−0.3000	0.9137	0.045*	
H14C	0.9685	−0.2134	0.8676	0.045*	
C15	0.6730 (7)	0.2668 (5)	0.8068 (4)	0.0193 (17)	
C16	0.5724 (7)	0.2661 (5)	0.8884 (4)	0.0265 (19)	
H16A	0.4857	0.3060	0.8734	0.040*	
H16B	0.6250	0.2930	0.9429	0.040*	
H16C	0.5424	0.1988	0.9009	0.040*	
C17	0.5873 (6)	0.2255 (4)	0.7230 (4)	0.0217 (17)	
H17A	0.5039	0.2681	0.7072	0.033*	
H17B	0.5518	0.1599	0.7372	0.033*	
H17C	0.6511	0.2219	0.6707	0.033*	
C18	0.7186 (7)	0.3708 (4)	0.7858 (4)	0.026 (2)	
H18A	0.6314	0.4105	0.7719	0.040*	
H18B	0.7807	0.3708	0.7326	0.040*	
H18C	0.7732	0.3981	0.8393	0.040*	
C21	0.5522 (7)	−0.0297 (6)	0.5894 (4)	0.0280 (18)	
H21A	0.5876	0.0317	0.5697	0.034*	
C22	0.3949 (9)	−0.1684 (6)	0.5652 (6)	0.084 (3)	
H22A	0.4454	−0.1893	0.6228	0.126*	
H22B	0.4086	−0.2177	0.5175	0.126*	
H22C	0.2903	−0.1607	0.5751	0.126*	
C23	0.4080 (7)	−0.0386 (7)	0.4445 (4)	0.057 (3)	
H23A	0.4425	0.0287	0.4377	0.086*	
H23B	0.3010	−0.0400	0.4378	0.086*	
H23C	0.4492	−0.0798	0.3970	0.086*	
C24	0.9571 (8)	0.0712 (5)	0.6335 (4)	0.0281 (19)	
H24A	1.0169	0.0208	0.6597	0.034*	
C25	0.9337 (7)	0.2131 (5)	0.5394 (5)	0.042 (2)	
H25A	0.8292	0.2014	0.5464	0.063*	
H25B	0.9532	0.2177	0.4738	0.063*	
H25C	0.9621	0.2744	0.5702	0.063*	
C26	1.1697 (7)	0.1266 (5)	0.5580 (4)	0.042 (2)	
H26A	1.2144	0.0720	0.5925	0.063*	
H26B	1.2207	0.1874	0.5751	0.063*	
H26C	1.1772	0.1151	0.4918	0.063*	

### Atomic displacement parameters (Å^2^)

**Table:**

	*U*^11^	*U*^22^	*U*^33^	*U*^12^	*U*^13^	*U*^23^
Sb1	0.0168 (3)	0.0299 (3)	0.0386 (3)	0.0007 (3)	−0.0009 (2)	0.0003 (3)
Sb2	0.0473 (4)	0.0535 (4)	0.0314 (3)	−0.0137 (3)	0.0082 (3)	−0.0042 (3)
Cu	0.0247 (5)	0.0150 (4)	0.0131 (4)	−0.0012 (5)	−0.0004 (3)	−0.0003 (5)
F1	0.081 (4)	0.043 (3)	0.149 (5)	0.000 (3)	−0.017 (4)	0.023 (3)
F2	0.051 (3)	0.109 (4)	0.050 (3)	0.010 (3)	0.003 (2)	−0.012 (3)
F3	0.060 (4)	0.174 (6)	0.071 (3)	−0.008 (4)	0.016 (3)	−0.074 (4)
F4	0.100 (5)	0.047 (3)	0.110 (5)	0.007 (3)	−0.021 (4)	0.038 (3)
F5	0.029 (2)	0.058 (4)	0.055 (2)	0.003 (2)	−0.0032 (17)	−0.001 (3)
F6	0.043 (3)	0.057 (4)	0.051 (3)	0.007 (2)	−0.003 (2)	−0.005 (2)
F11	0.092 (5)	0.070 (4)	0.078 (4)	0.020 (3)	0.047 (3)	0.034 (3)
F12	0.064 (3)	0.129 (5)	0.089 (4)	0.004 (4)	−0.034 (3)	−0.037 (4)
F13	0.063 (4)	0.092 (4)	0.084 (4)	−0.004 (3)	0.017 (3)	−0.036 (3)
F14	0.088 (4)	0.058 (3)	0.061 (3)	0.008 (3)	0.008 (3)	−0.011 (3)
F15	0.106 (5)	0.115 (5)	0.079 (4)	−0.056 (4)	0.039 (3)	0.012 (3)
F16	0.065 (3)	0.082 (4)	0.074 (3)	−0.013 (3)	−0.015 (3)	−0.008 (3)
O1	0.043 (3)	0.013 (3)	0.023 (3)	−0.011 (2)	0.008 (2)	0.004 (2)
O2	0.040 (3)	0.016 (3)	0.023 (3)	−0.009 (2)	−0.011 (2)	0.004 (2)
O11	0.038 (3)	0.024 (3)	0.015 (3)	−0.006 (2)	−0.019 (2)	0.004 (2)
O12	0.020 (3)	0.023 (3)	0.018 (3)	0.001 (2)	0.007 (2)	0.006 (2)
N11	0.038 (4)	0.065 (6)	0.026 (4)	0.007 (4)	−0.012 (3)	−0.021 (4)
N12	0.015 (3)	0.027 (4)	0.034 (4)	0.004 (3)	0.005 (3)	0.004 (3)
C1	0.019 (4)	0.015 (4)	0.017 (4)	−0.004 (3)	0.003 (3)	0.001 (3)
C2	0.025 (5)	0.019 (4)	0.027 (5)	−0.006 (4)	0.002 (4)	0.006 (4)
C4	0.020 (3)	0.014 (3)	0.014 (3)	0.000 (4)	−0.002 (3)	0.006 (4)
C6	0.041 (5)	0.006 (4)	0.027 (4)	−0.007 (3)	−0.012 (4)	0.005 (3)
C7	0.019 (4)	0.017 (4)	0.010 (4)	−0.004 (3)	−0.002 (3)	0.001 (3)
C8	0.045 (5)	0.027 (6)	0.020 (4)	−0.002 (4)	0.010 (3)	−0.012 (3)
C9	0.038 (4)	0.019 (4)	0.026 (4)	−0.002 (4)	−0.007 (3)	−0.005 (4)
C11	0.031 (5)	0.015 (4)	0.023 (5)	0.009 (4)	0.001 (4)	−0.003 (4)
C12	0.030 (5)	0.036 (5)	0.023 (4)	0.005 (4)	0.008 (4)	−0.004 (4)
C13	0.053 (6)	0.030 (5)	0.051 (6)	0.001 (5)	−0.002 (5)	−0.005 (5)
C14	0.028 (5)	0.032 (5)	0.028 (5)	−0.004 (4)	−0.014 (4)	0.003 (4)
C15	0.024 (5)	0.021 (4)	0.013 (4)	0.003 (4)	−0.002 (3)	−0.001 (3)
C16	0.031 (5)	0.031 (5)	0.017 (4)	0.012 (4)	−0.004 (4)	−0.005 (4)
C17	0.020 (4)	0.026 (4)	0.019 (4)	0.007 (3)	0.000 (3)	0.003 (3)
C18	0.044 (5)	0.015 (4)	0.020 (4)	−0.002 (4)	−0.006 (4)	−0.004 (4)
C21	0.020 (4)	0.029 (5)	0.035 (4)	−0.005 (4)	0.010 (3)	−0.010 (4)
C22	0.082 (8)	0.087 (8)	0.080 (8)	−0.037 (7)	−0.030 (6)	−0.005 (7)
C23	0.052 (5)	0.092 (7)	0.026 (4)	0.016 (6)	−0.017 (4)	−0.008 (6)
C24	0.031 (5)	0.026 (5)	0.028 (4)	−0.006 (4)	0.006 (4)	0.005 (4)
C25	0.030 (5)	0.040 (5)	0.056 (6)	−0.001 (4)	0.006 (4)	0.018 (4)
C26	0.022 (5)	0.053 (5)	0.052 (5)	−0.004 (4)	0.005 (4)	0.005 (5)

### Geometric parameters (Å, °)

**Table:**

Sb1—F4	1.834 (4)	C7—H7A	1.0000
Sb1—F5	1.844 (3)	C8—H8A	0.9800
Sb1—F3	1.847 (4)	C8—H8B	0.9800
Sb1—F6	1.848 (4)	C8—H8C	0.9800
Sb1—F2	1.853 (4)	C9—H9A	0.9800
Sb1—F1	1.859 (4)	C9—H9B	0.9800
Sb2—F11	1.840 (4)	C9—H9C	0.9800
Sb2—F15	1.844 (4)	C11—C14	1.509 (8)
Sb2—F16	1.862 (4)	C11—C13	1.528 (9)
Sb2—F12	1.865 (4)	C11—C12	1.534 (8)
Sb2—F13	1.868 (5)	C12—H12A	0.9800
Sb2—F14	1.879 (4)	C12—H12B	0.9800
Cu—N1	1.929 (5)	C12—H12C	0.9800
Cu—N2	1.919 (5)	C13—H13A	0.9800
Cu—O11	1.926 (4)	C13—H13B	0.9800
Cu—O12	1.951 (4)	C13—H13C	0.9800
O1—C3	1.322 (6)	C14—H14A	0.9800
O1—C2	1.450 (7)	C14—H14B	0.9800
O2—C5	1.331 (7)	C14—H14C	0.9800
O2—C6	1.461 (6)	C15—C18	1.518 (8)
O11—C21	1.249 (7)	C15—C17	1.528 (8)
O12—C24	1.277 (7)	C15—C16	1.534 (8)
N1—C3	1.288 (7)	C16—H16A	0.9800
N1—C1	1.518 (7)	C16—H16B	0.9800
N2—C5	1.283 (7)	C16—H16C	0.9800
N2—C7	1.516 (7)	C17—H17A	0.9800
N11—C21	1.314 (7)	C17—H17B	0.9800
N11—C22	1.453 (9)	C17—H17C	0.9800
N11—C23	1.471 (7)	C18—H18A	0.9800
N12—C24	1.303 (7)	C18—H18B	0.9800
N12—C25	1.418 (7)	C18—H18C	0.9800
N12—C26	1.464 (7)	C21—H21A	0.9500
C1—C11	1.532 (8)	C22—H22A	0.9800
C1—C2	1.536 (8)	C22—H22B	0.9800
C1—H1A	1.0000	C22—H22C	0.9800
C2—H2A	0.9900	C23—H23A	0.9800
C2—H2B	0.9900	C23—H23B	0.9800
C3—C4	1.532 (8)	C23—H23C	0.9800
C4—C5	1.486 (8)	C24—H24A	0.9500
C4—C9	1.545 (7)	C25—H25A	0.9800
C4—C8	1.556 (7)	C25—H25B	0.9800
C6—C7	1.546 (8)	C25—H25C	0.9800
C6—H6A	0.9900	C26—H26A	0.9800
C6—H6B	0.9900	C26—H26B	0.9800
C7—C15	1.547 (8)	C26—H26C	0.9800
			
F4—Sb1—F5	92.1 (2)	C4—C8—H8B	109.5
F4—Sb1—F3	91.0 (3)	H8A—C8—H8B	109.5
F5—Sb1—F3	91.25 (17)	C4—C8—H8C	109.5
F4—Sb1—F6	89.3 (2)	H8A—C8—H8C	109.5
F5—Sb1—F6	178.25 (17)	H8B—C8—H8C	109.5
F3—Sb1—F6	89.81 (18)	C4—C9—H9A	109.5
F4—Sb1—F2	90.3 (2)	C4—C9—H9B	109.5
F5—Sb1—F2	88.80 (16)	H9A—C9—H9B	109.5
F3—Sb1—F2	178.8 (3)	C4—C9—H9C	109.5
F6—Sb1—F2	90.10 (17)	H9A—C9—H9C	109.5
F4—Sb1—F1	179.0 (2)	H9B—C9—H9C	109.5
F5—Sb1—F1	87.15 (19)	C14—C11—C13	110.1 (6)
F3—Sb1—F1	89.7 (3)	C14—C11—C1	111.7 (5)
F6—Sb1—F1	91.5 (2)	C13—C11—C1	106.1 (6)
F2—Sb1—F1	89.1 (2)	C14—C11—C12	109.1 (6)
F11—Sb2—F15	178.4 (2)	C13—C11—C12	109.3 (5)
F11—Sb2—F16	89.0 (2)	C1—C11—C12	110.5 (5)
F15—Sb2—F16	91.6 (2)	C11—C12—H12A	109.5
F11—Sb2—F12	89.2 (2)	C11—C12—H12B	109.5
F15—Sb2—F12	90.2 (2)	H12A—C12—H12B	109.5
F16—Sb2—F12	177.7 (2)	C11—C12—H12C	109.5
F11—Sb2—F13	90.3 (2)	H12A—C12—H12C	109.5
F15—Sb2—F13	91.1 (2)	H12B—C12—H12C	109.5
F16—Sb2—F13	90.0 (2)	C11—C13—H13A	109.5
F12—Sb2—F13	88.5 (2)	C11—C13—H13B	109.5
F11—Sb2—F14	88.66 (18)	H13A—C13—H13B	109.5
F15—Sb2—F14	89.9 (2)	C11—C13—H13C	109.5
F16—Sb2—F14	90.5 (2)	H13A—C13—H13C	109.5
F12—Sb2—F14	90.9 (2)	H13B—C13—H13C	109.5
F13—Sb2—F14	178.9 (2)	C11—C14—H14A	109.5
N2—Cu—O11	153.82 (19)	C11—C14—H14B	109.5
N2—Cu—N1	92.92 (17)	H14A—C14—H14B	109.5
O11—Cu—N1	91.37 (19)	C11—C14—H14C	109.5
N2—Cu—O12	95.00 (19)	H14A—C14—H14C	109.5
O11—Cu—O12	91.51 (17)	H14B—C14—H14C	109.5
N1—Cu—O12	155.84 (18)	C18—C15—C17	108.8 (5)
C3—O1—C2	106.1 (5)	C18—C15—C16	109.9 (5)
C5—O2—C6	106.2 (5)	C17—C15—C16	108.0 (6)
C21—O11—Cu	125.9 (5)	C18—C15—C7	108.3 (5)
C24—O12—Cu	126.2 (4)	C17—C15—C7	110.4 (5)
C3—N1—C1	107.0 (5)	C16—C15—C7	111.3 (5)
C3—N1—Cu	126.8 (4)	C15—C16—H16A	109.5
C1—N1—Cu	125.5 (4)	C15—C16—H16B	109.5
C5—N2—C7	106.6 (5)	H16A—C16—H16B	109.5
C5—N2—Cu	127.2 (4)	C15—C16—H16C	109.5
C7—N2—Cu	126.1 (4)	H16A—C16—H16C	109.5
C21—N11—C22	120.1 (6)	H16B—C16—H16C	109.5
C21—N11—C23	122.1 (7)	C15—C17—H17A	109.5
C22—N11—C23	117.8 (6)	C15—C17—H17B	109.5
C24—N12—C25	122.0 (6)	H17A—C17—H17B	109.5
C24—N12—C26	120.2 (6)	C15—C17—H17C	109.5
C25—N12—C26	117.8 (5)	H17A—C17—H17C	109.5
N1—C1—C11	113.0 (5)	H17B—C17—H17C	109.5
N1—C1—C2	100.5 (5)	C15—C18—H18A	109.5
C11—C1—C2	115.7 (5)	C15—C18—H18B	109.5
N1—C1—H1A	109.1	H18A—C18—H18B	109.5
C11—C1—H1A	109.1	C15—C18—H18C	109.5
C2—C1—H1A	109.1	H18A—C18—H18C	109.5
O1—C2—C1	104.9 (5)	H18B—C18—H18C	109.5
O1—C2—H2A	110.8	O11—C21—N11	123.1 (7)
C1—C2—H2A	110.8	O11—C21—H21A	118.5
O1—C2—H2B	110.8	N11—C21—H21A	118.5
C1—C2—H2B	110.8	N11—C22—H22A	109.5
H2A—C2—H2B	108.8	N11—C22—H22B	109.5
N1—C3—O1	117.8 (6)	H22A—C22—H22B	109.5
N1—C3—C4	128.0 (6)	N11—C22—H22C	109.5
O1—C3—C4	114.1 (5)	H22A—C22—H22C	109.5
C5—C4—C3	113.2 (4)	H22B—C22—H22C	109.5
C5—C4—C9	111.2 (5)	N11—C23—H23A	109.5
C3—C4—C9	107.6 (5)	N11—C23—H23B	109.5
C5—C4—C8	108.1 (5)	H23A—C23—H23B	109.5
C3—C4—C8	107.0 (5)	N11—C23—H23C	109.5
C9—C4—C8	109.5 (4)	H23A—C23—H23C	109.5
N2—C5—O2	117.8 (6)	H23B—C23—H23C	109.5
N2—C5—C4	129.8 (6)	O12—C24—N12	122.4 (7)
O2—C5—C4	112.4 (5)	O12—C24—H24A	118.8
O2—C6—C7	103.3 (5)	N12—C24—H24A	118.8
O2—C6—H6A	111.1	N12—C25—H25A	109.5
C7—C6—H6A	111.1	N12—C25—H25B	109.5
O2—C6—H6B	111.1	H25A—C25—H25B	109.5
C7—C6—H6B	111.1	N12—C25—H25C	109.5
H6A—C6—H6B	109.1	H25A—C25—H25C	109.5
N2—C7—C6	100.7 (4)	H25B—C25—H25C	109.5
N2—C7—C15	112.5 (5)	N12—C26—H26A	109.5
C6—C7—C15	116.3 (5)	N12—C26—H26B	109.5
N2—C7—H7A	108.9	H26A—C26—H26B	109.5
C6—C7—H7A	108.9	N12—C26—H26C	109.5
C15—C7—H7A	108.9	H26A—C26—H26C	109.5
C4—C8—H8A	109.5	H26B—C26—H26C	109.5
			
N2—Cu—O11—C21	74.3 (6)	C7—N2—C5—O2	−7.8 (8)
N1—Cu—O11—C21	173.7 (5)	Cu—N2—C5—O2	168.6 (4)
O12—Cu—O11—C21	−30.3 (5)	C7—N2—C5—C4	171.1 (6)
N2—Cu—O12—C24	84.8 (5)	Cu—N2—C5—C4	−12.5 (10)
O11—Cu—O12—C24	−120.6 (5)	C6—O2—C5—N2	−8.0 (8)
N1—Cu—O12—C24	−23.9 (8)	C6—O2—C5—C4	172.9 (5)
N2—Cu—N1—C3	9.6 (6)	C3—C4—C5—N2	7.2 (10)
O11—Cu—N1—C3	−144.6 (5)	C9—C4—C5—N2	128.5 (7)
O12—Cu—N1—C3	118.7 (6)	C8—C4—C5—N2	−111.2 (7)
N2—Cu—N1—C1	178.9 (4)	C3—C4—C5—O2	−173.9 (5)
O11—Cu—N1—C1	24.8 (5)	C9—C4—C5—O2	−52.5 (7)
O12—Cu—N1—C1	−72.0 (7)	C8—C4—C5—O2	67.8 (6)
O11—Cu—N2—C5	103.2 (6)	C5—O2—C6—C7	19.3 (6)
N1—Cu—N2—C5	4.1 (6)	C5—N2—C7—C6	18.7 (6)
O12—Cu—N2—C5	−153.1 (5)	Cu—N2—C7—C6	−157.7 (4)
O11—Cu—N2—C7	−81.1 (6)	C5—N2—C7—C15	−105.8 (6)
N1—Cu—N2—C7	179.8 (4)	Cu—N2—C7—C15	77.7 (6)
O12—Cu—N2—C7	22.6 (5)	O2—C6—C7—N2	−22.4 (6)
C3—N1—C1—C11	−110.4 (6)	O2—C6—C7—C15	99.5 (6)
Cu—N1—C1—C11	78.6 (6)	N1—C1—C11—C14	64.9 (7)
C3—N1—C1—C2	13.5 (6)	C2—C1—C11—C14	−50.2 (8)
Cu—N1—C1—C2	−157.5 (4)	N1—C1—C11—C13	−175.2 (5)
C3—O1—C2—C1	17.6 (6)	C2—C1—C11—C13	69.7 (7)
N1—C1—C2—O1	−18.4 (6)	N1—C1—C11—C12	−56.9 (7)
C11—C1—C2—O1	103.7 (6)	C2—C1—C11—C12	−172.0 (5)
C1—N1—C3—O1	−3.2 (7)	N2—C7—C15—C18	−176.6 (5)
Cu—N1—C3—O1	167.7 (4)	C6—C7—C15—C18	67.9 (7)
C1—N1—C3—C4	172.4 (6)	N2—C7—C15—C17	−57.5 (6)
Cu—N1—C3—C4	−16.7 (9)	C6—C7—C15—C17	−173.0 (5)
C2—O1—C3—N1	−9.6 (7)	N2—C7—C15—C16	62.4 (7)
C2—O1—C3—C4	174.2 (5)	C6—C7—C15—C16	−53.1 (8)
N1—C3—C4—C5	8.3 (9)	Cu—O11—C21—N11	−175.2 (4)
O1—C3—C4—C5	−175.9 (5)	C22—N11—C21—O11	1.8 (10)
N1—C3—C4—C9	−115.0 (7)	C23—N11—C21—O11	−175.3 (5)
O1—C3—C4—C9	60.7 (6)	Cu—O12—C24—N12	−165.4 (4)
N1—C3—C4—C8	127.3 (6)	C25—N12—C24—O12	1.2 (10)
O1—C3—C4—C8	−56.9 (7)	C26—N12—C24—O12	−178.2 (6)

### Hydrogen-bond geometry (Å, °)

**Table:**

*D*—H···*A*	*D*—H	H···*A*	*D*···*A*	*D*—H···*A*
C12—H12A···O11	0.98	2.58	3.204 (8)	122
C17—H17C···O12	0.98	2.58	3.189 (7)	120
C25—H25C···F13^i^	0.98	2.55	3.418 (8)	148
C26—H26B···F11^i^	0.98	2.53	3.427 (8)	152

Symmetry codes: (i) −*x*+2, *y*+1/2, −*z*+1.
